# How are Medical Institutions Supporting the Well-being of Undergraduate Students? A Scoping Review

**DOI:** 10.1080/10872981.2022.2133986

**Published:** 2022-10-21

**Authors:** Shakila Mohmand, Sasha Monteiro, Leslie Solomonian

**Affiliations:** Research Department, Canadian College of Naturopathic Medicine, Toronto, ON, Canada

**Keywords:** Burnout, medical education, medical institution, medical students, resilience, stress, well-being, wellness strategies

## Abstract

**Introduction:**

Medical students experience significant stress and impacts on mood due to multiple factors. Unmitigated stress impacts both physical and mental health while increasing the risk of unethical behavior. It is important for medical institutions to identify strategies that effectively reduce perceived stress and improve the well-being of their students.

**Methods:**

The authors undertook a scoping review of the literature to identify strategies implemented by medical educational programs to improve the well-being of medical students.

**Results:**

Of 1068 articles identified, 19 studies met the inclusion criteria. Interventions were categorized as mindfulness-based programs, reflection groups, curriculum changes, and ‘miscellaneous.’ All studies assessed outcomes of student stress/resilience, as well as additional domains including academic performance, mental health, and interpersonal skills. Some also assessed the acceptability of the intervention to students.

**Conclusions:**

Despite the heterogeneity of interventions and outcome measures, a clear theme emerged that institutionally-provided strategies to promote student well-being tend to be effective when students opt into the program. It was noted that adding mandatory content or activities to a medical program without creating adequate space or support for it can have the opposite effect. Further high quality intervention studies involving randomization, blinding and rigorous controls are warranted.

## Introduction

Medical students demonstrate a higher degree of stress, depression and burnout compared to other student groups. Reported levels of stress within medical student populations range between 25 to 75% [1]. Persistently high levels of stress can be maladaptive, leading to mood changes and burnout [2,3]. [4] found that 20% of medical students reported problematic drinking due to stress and anxiety. Burnout rates among medical students in North America may be as high as 49% with suicide a potential serious consequence [5]. Burnout in medical school may also increase the risk of unethical behaviour, and compromise patient care [6] in part by increasing depersonalisation, which can hinder the ability to build trust and therapeutic relationships [7]. The educational experience plays a large role in the stress experience by medical students [8]. Medical educational institutions have an opportunity to implement strategies that reduce intolerable sources of stress, and to build the capacity of students to demonstrate resilience.

There are a number of factors that contribute to the decreased well-being of medical students including intrinsic factors such as high personal standards correlated with perfectionism and neuroticism [9], and extrinsic factors such as financial loans and uncertain career choices [5,10,11]. [12] described a conceptual model of medical student well-being, in which they described internal and external factors that impact an individual’s ‘coping reservoir.’ They also identified implications of this reservoir, ranging from enhanced resilience to burnout.

**Figure 1.**
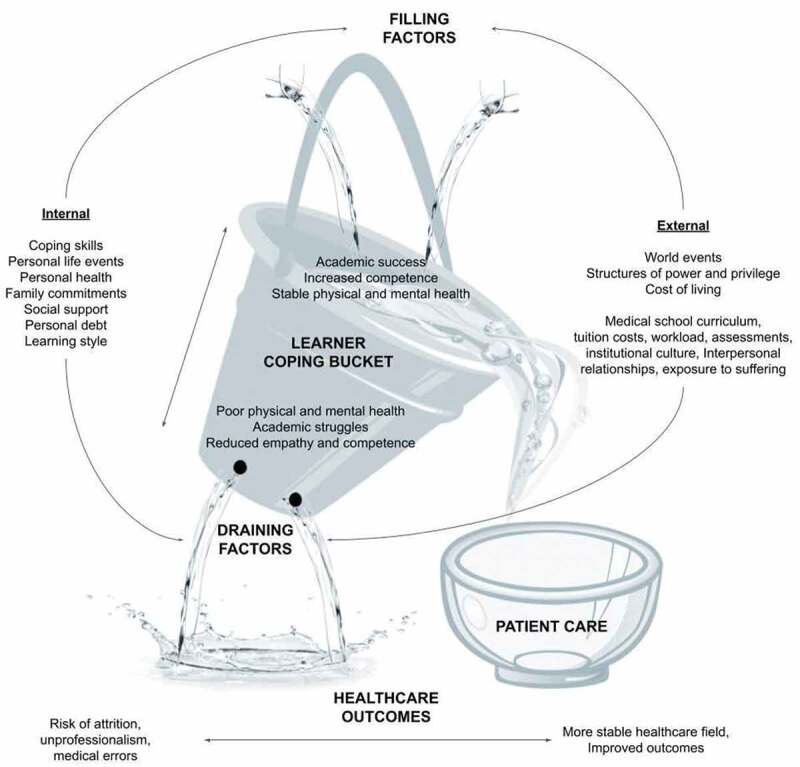
A ‘bucket’ conceptual framework for medical student resilience (courtesy of authors, with acknowledgement to Dunn et al). A conceptual framework for the review, building on the ‘coping reservoir’ model proposed by Dunn et al. Internal and external factors – including the program offered by the educational institution – can contribute to the resilience of medical students, or can cause draining of the coping reservoir. This leads to burnout, poor mental and physical health, and potentially poorer patient care.

The image of a leaking bucket is a metaphor that illustrates the ways in which factors affect the ability to cope with stress (Figure 1). Positive (or ‘filling’) and negative (or ‘draining’) inputs to a student’s ‘coping bucket’ can come from a variety of sources. When considering the implications of attempting to provide healthcare with an ‘empty’ bucket, medical institutions have an opportunity – and arguably an obligation – to enhance positive contributions of education programs, mitigate negative influences, and provide mechanisms to build strategies of resilience among trainees. This review explores strategies that have been implemented by medical institutions to improve the well-being of students.

## Methods

The authors selected a scoping instead of a systematic review as being the most appropriate methodology to explore this topic. Due to the heterogeneity of interventions and outcome measures that were noted in a literature scan, it was deemed to be more useful to describe what has been done and identify themes, rather than attempt to answer a specific question about which exact approach is most recommended [13]. The methodology followed the framework presented by [14] for the conduct of scoping reviews.

### Aim and objectives

This review aimed to identify interventions that have been used by medical institutions to reduce medical student burnout and improve well-being. The goal is to inform the design and implementation of strategies to promote well-being within medical institutions.

### Search Strategy

Relevant articles were identified by searching the PubMed database from the date of inception to 1 December 2021. The combination of search terms that were utilized were as follows: ‘Well-being + medical students + strategies’, ‘Well-being + medical students + tools’, ‘Well-being + medical students + stress management’, ‘Well-being + medical students + coping’, ‘Well-being + medical students + support’.

### Selection Criteria

Inclusion criteria included English language interventional studies involving undergraduate medical students, with clearly defined well-being focused outcome measures. Randomized and nonrandomized as well as controlled and non-controlled studies were included. Both quantitative and qualitative designs were included. Systematic reviews, narrative reviews, and studies lacking a clear intervention strategy or outcome assessment were excluded. Studies of mixed populations were excluded if results specific to medical students were not clearly distinguished.

### Data organization

Data was screened in three rounds by two independent reviewers. The first round of selection consisted of a title screen for publications related to medical student well-being. Relevant titles were documented in a spreadsheet and screened for duplicates which were removed. The second round involved screening abstracts to determine if the objective of the papers aligned with the objective of the review. The final screening was a full text review to ensure that the included papers stated clear interventions and outcomes focused specifically on medical students. Any disagreement was resolved by consensus between the two reviewers. Data extraction was completed using piloted extraction templates for studies. Complete study data was extracted by one reviewer.

### Data Analysis

Studies were characterized by intervention type, study design, and outcome measures. Statistical pooling was not performed given the nature and objective of the review. Risk of bias analysis was performed using the ‘Study of Quality Assessment Tools’ developed by the National Heart, Blood, and Lung.

## Results

**Figure 2.**
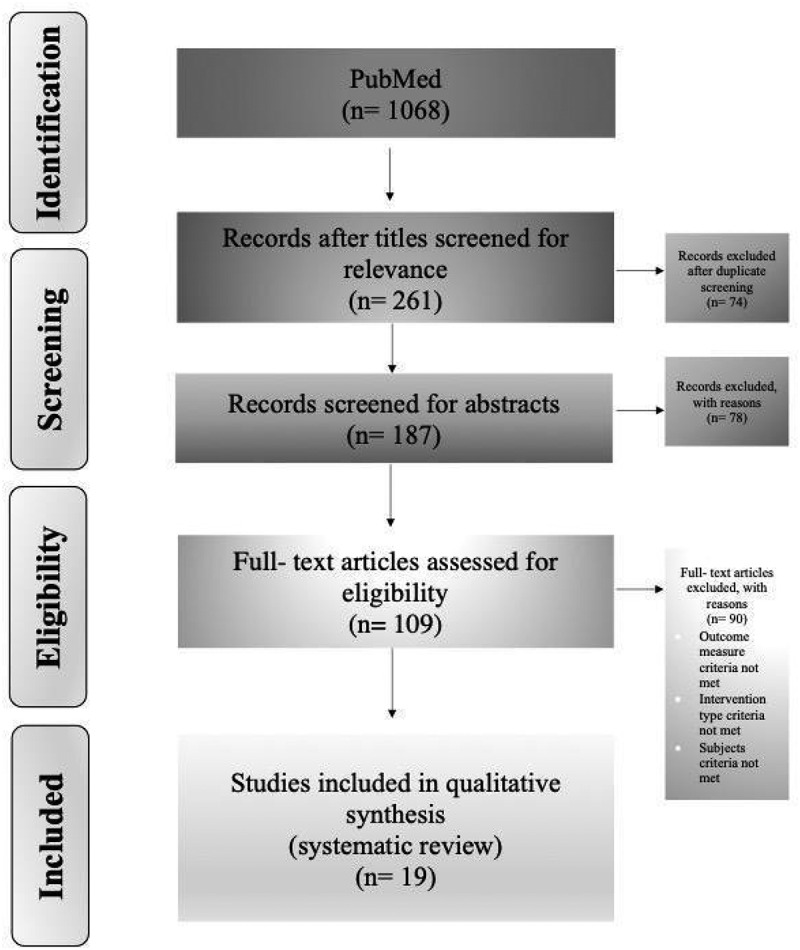
Selection of studies included in the scoping review PRISMA diagram illustrating the identification and selection of articles.

A total of 1068 references were identified, of which 109 were potentially eligible after title and abstract screening. After full text review, 19 studies met the *a priori* inclusion criteria (see PRISMA flowchart [Figure 2]). Details of these studies are described in Table 1 and 2, as well as summarized below.

**Table 1.** Summary of studies reviewed.

Mind-Body or Mindfulness Based Programs			
Author [Year)	Country of Origin	Participants	Quality rating
15	USA	n = 27	Fair
27	USA	n = 104	Fair
23	Canada	n = 30	Fair
de Vibe, M et al. [21,22]	Norway	n = 144	Good
25	USA	n = 66	Fair
17	USA	n = 28	Fair
16	USA	n = 24	Good
24	UK	n = 57	Fair
26	Israel	n = 3	Fair
18	USA	n = 24	Fair
19	China	n = 50	Fair
20	USA	n = 45	Good
Miscellaneous Elective Courses			
28	USA	n = 28	Fair
29	Canada	n = 18	Good
30	Ireland	n = 74	Fair
Reflection Groups			
32,	UK	n = 1827	Fair
31	USA	n = 30	Fair
Curriculum Changes			
33	Kuwait	n = 345	Fair
34	USA	n = 350–353	Fair

Abbreviation: RCT, randomized control trial.

**Table 2.** Studies included in review.

Mind-Body or Mindfulness Based Programs						
Author [Year)	Design	Participants	Intervention	Control	Outcome Measures	Results
15	NRNCT	1st and 2nd year medical students n = 27	11 weekly elective “embodied health” course	N/A	Jefferson Scale of Physician Empathy Cohen’s Perceived Stress Scale Self-Regulation Questionnaire Self-Compassion Scale	Increase in self-regulation and self-compassion Increase in empathy
27	NRNCT	3rd and 4th year medical students n = 104	Mandatory Brief Behavioral Intervention Program [BBIP]	N/A	General Well-Being Scale	Decreased stress; decreased anxiety; improved positive well-being.
23	RCT	1st and 2nd year medical students n = 30	Eight-week elective mindfulness meditation program	Waitlist n = 270	Self-reported psychological distress, empathy, self-compassion, mindfulness, altruism, professionalism, and program satisfaction	High level of satisfaction; positive impact on academic performance; positive impact on clinical performance.
de Vibe, M et al. [21,22]	RCT	n = 144	Seven week elective mindfulness‐based stress reduction program	Control n = 144	General Health Questionnaire Maslach Burnout Inventory Perceived Medical institution Stress Scale Subjective Well‐being Scale Five Facet Mindfulness Questionnaire	Moderate reduction in mental distress; small improvement in subjective well‐being and ‘non‐judging;’ significant reduction in study stress for female students
25	NRNCT	2 cohorts of 1st year medical students n = 66	Curricular [mandatory] mindfulness-based stress management course	N/A	Maslach Burnout Inventory Medical Outcomes Study Short Form Perceived Stress Scale Connor Davidson Resilience Scale Happiness and Gratitude Scale	Decrease in mean mental QOL and happiness declined; increased stress; decline in cognitive and emotive empathy; no statistically significant changes in burnout or resilience.
17	NRCT	1st and 2nd year medical students n = 28	Eleven weekly elective mind-body sessions	Control n = 24	Distress tolerance scale Cognitive and affective mindfulness scale revised Perceived stress scale positive affect and negative affect schedule	Improved tolerance of affective distress; improvement in psychological symptoms associated with improvements in distress tolerance; positive satisfaction with program
16	NRCT	1st year medical students n = 24	Eleven weekly elective mind-body skills sessions	Control n = 22	Blood cortisol, DHEA-S, testosterone, and secretory immunoglobulin	Significant reduction in mean morning salivary cortisol, DHEA-S and testosterone; no change in sIgA levels; stability of hormonal levels throughout the semester compared to increases in controls
24	Qualitative	1st-3rd year medical students n = 57	Eight-week elective mindfulness training program	N/A	Open- ended text survey and qualitative interviews regarding well- being, coping reserve and professional development	Themes: greater awareness of thoughts and feelings; greater sense of control and resilience; improved ability to manage workload; greater acceptance of limitations as learners; improved empathy; improved communication skills
26	NRNCT	6th year medical students n = 3	2- hr workshop Mind-Body Medicine Skills combined with interactive reflective writing Reflection 1-month post-workshop	N/A	Professional Quality of Life measure Questionnaire evaluating understanding of professional burnout, resilience and perception on preparation for applying workshop techniques	Participants reported a better understanding of professional burnout/ resilience and felt better able to use meditation and reflective writing as coping tools
18	Mixed	1st and 2nd year medical students n = 24	Eleven week elective mind–body medicine course	N/A	Freiburg Mindfulness Inventory [FMI] Perceived Stress Scale Open text survey items Satisfaction	Improved average mindfulness scores No changes to perceived stress scale Positive satisfaction. Improvements in mindfulness, relationships with peers, and having a safe place to receive support.
19	RCT	1st year medical students n = 50	Five weekly elective well-being therapy sessions	Control n = 51	Psychological well-being, adaptation, anxiety and depression	Improvements in psychological well-being and adaptation; alleviation in symptoms of anxiety and depression
20	RCT	1st- 4th year medical students n = 45	Elective daily use [10–20 min] of audio-guided mindfulness meditation program	Waitlist n = 43	Perceived Stress Scale Five-Facet Mindfulness Questionnaire General Well-Being Schedule	Significant decrease in perceived stress; significant increase in general well-being
Miscellaneous Elective Courses						
28	NRNCT	1st and 2nd year medical students n = 28	Equine- assisted course; seven sessions over 2 months	N/A	Beck Depression Inventory-II Stress Factors adapted scale Perceived Stress Scale	Significantly reduced perceived stress, depression, stress severity, and stress frequency
29	Qualitative	1st year medical students n = 18	Six theater based special study modules	N/A	Semi-structured focus groups	3 thematic areas identified: 1] fun/enjoyment/relaxation 2) enhanced relationship connections 3) personal growth/resilience
30	Mixed	1st and 2nd year medical students n = 74	Six weekly elective exercise-based workshops	No control	Beliefs about exercise as medicine; level of physical activity; state of health and well-being; concentration WHO-5 Well-Being Index Sleep scale Loneliness scale Satisfaction with program	Increased well-being, improved sleep, decreased loneliness, increased physical activity; improvement in perception of the importance of exercise as a treatment modality; strong satisfaction
Reflection Groups						
32	mixed	3rd year medical students n = 1827	12 weekly mandatory small group clinical debrief discussions	N/A	Online surveys	Positive satisfaction Students appreciated safe environments, the session structure, facilitator role modeling, transitional support and processing of emotional experiences.
31	Mixed	1st and 2nd year medical students n = 30	Biweekly elective reflection groups	N/A	Modified Emotional Self-Awareness Scale, modified Interpersonal Fulfillment Index, and Revised UCLA Loneliness Scale	Improved well-being, enhanced self-awareness, improved ability to empathize, increased sense of connection
Curriculum Changes						
33	NRNCT	5th and 6th year dental students n = 345	Subject-based curriculum vs. case-based integrated curriculum	N/A	A modified Dental Environment Stress questionnaire	Increased stress levels
34	NRCT	1st year medical students n = 350–353	Changes to curriculum (Pass/ fail for preclinical courses; reduction of contact hours in first two years of study; longitudinal electives; creation of learning communities; addition of a mandatory resilience and mindfulness program	Students in Class of 2011 and 2012, preceding curricular changes	Center for Epidemiological Studies Depression Scale, Spielberger State-Trait Anxiety Inventory, Perceived Stress Scale, Perceived Cohesion Scale Association of American Medical Colleges’ Graduation Questionnaire	Increased satisfaction with the program For the classes of 2011 and 2012, the mean Step 1 score was 224.5; for the class of 2013, 227; and for the class of 2014, 230. The mean score for the class of 2014 was significantly higher (p = 0.002) than that for the classes of 2011 and 2012.

Abbreviations: RCT: randomized control trial; NRCT: non-randomized control trial; NRNCT: non-randomized non-controlled trial

### Interventions used

A range of interventions were noted. Most prevalent were mindfulness-based programs [n = 12], although other approaches to well-being were also evident, including a theatre elective, equine therapy, and systematic encouragement of physical activity. Some institutions assessed the impact of curriculum changes [n = 2], and reflection groups [n = 2]. Most of the interventions were longitudinal programs [n = 15], while the remainder consisted of individual workshops.

### Study designs

Study designs included randomized [n = 4] and non-randomized [n = 3] control trials, non-controlled before/after studies [n = 5], mixed methods [n = 5], and qualitative [n = 2]. Five studies reported on mandatory or school-wide interventions, while the remainder reflected elective participation.

### Outcomes assessed

Studies assessed outcomes within a variety of domains. Outcomes related to ‘stress’ or ‘resilience’ were noted in every study. Additional outcome measures identified included mental health [n = 6], academic performance [n = 2], interpersonal dynamics [n = 8], and acceptability of the intervention [n = 8]. A variety of validated rating tools and Likert scales were used for quantitative assessments. Qualitative and mixed methods studies collected data through interviews, focus groups, open-text surveys, and written reflections.

**Figure 3.**
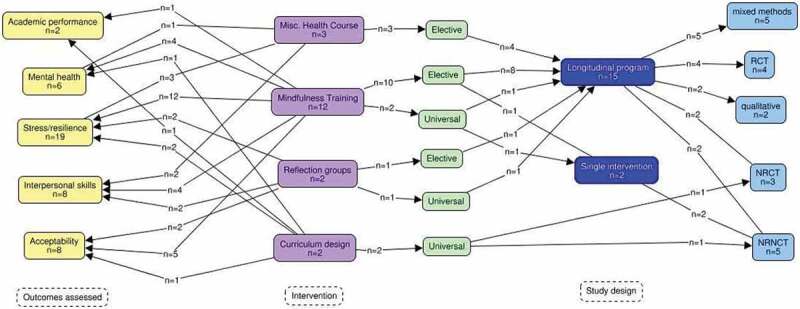
A mapping of studies reviewed by outcomes (yellow], intervention (purple/green/dark blue), and study design [light blue). A mapping of reviewed studies, describing categories of interventions, study design, and outcomes assessed.

A map of the relationships between interventions, study designs, and outcome measures is shown in Figure 3.

Most studies demonstrated an improvement in outcome measures associated with the intervention. The only study that did not yield an improvement was the only one in which the intervention consisted of a mandatory course added to the existing curriculum.

### Summary of Studies

#### Mindfulness and Mind-Body Skills Training

Most mindfulness-based interventions were elective. Some unfolded over a series of weeks, while others were delivered in one session.

[15] assessed the effect of an 11-week mind-body focused elective course which included practices such as yoga and meditation. Participants reported an overall decrease in perceived stress, as well as improvements in self-regulation, self-compassion, and empathy. Students also reported increased skills of mindfulness and stress management. [16] tracked the physiological response of medical students to an 11-week mind- body medicine elective which included autogenic training, biofeedback, imagery, journaling, and meditation. Mean morning salivary cortisol, dehydroepiandrosterone sulfate [DHEA-S] and testosterone all significantly decreased post-intervention compared to controls. This quantitative evidence of a positive impact on the hypothalamic-pituitary-adrenal axis was positively correlated with stress management skills reflected in questionnaire responses. [17] also assessed the effects of an 11- week program focused on mind-body skills including biofeedback, guided imagery, relaxation, meditation, breathing exercises, and autogenic training. They concluded that the program led to improvements in the ability of students to tolerate distress compared to controls and improved affect. Students reported positive views of the program and recommended it to other students. [18] assessed a resilience focused mind-body medicine elective which exposed students to techniques of mindfulness, biofeedback, art, and journaling in small group settings. 95.8% of participants noted the value of the intervention and cited that the domains most significantly impacted were mindfulness skills, relationships with peers, and feelings of safety and support.

[19] conducted a single-blind randomized control trial to study the effect of five 2-hour group mindfulness sessions reinforced by assigned homework to promote well-being among 101 first year medical students. Results showed a non-significant tendency toward reduced depression and anxiety compared to controls. [20] demonstrated that audio-guided mindfulness meditation led to significantly decreased levels of perceived stress and improved reports of well-being compared to controls.

[21] assessed group reflections on a 7- week program of physical and mental exercises to promote stress management skills and mindful communication. Positive impacts were seen in subjective well-being and mindfulness skills, as measured by the General Health Questionnaire, Maslach Burnout Inventory, Perceived Medical institution School Stress Scale, Subjective Well-being Scale and the Five Facet Mindfulness Questionnaire. Results were sustained after 6 years as students who had participated reported increased well-being, greater increases in the trajectory of dispositional mindfulness and problem-focused coping, and a greater decrease in the trajectory of avoidance-focused coping compared to controls [22]. This was the only study in the review to include a multi-year follow-up.

[23] assessed the effectiveness of an 8-week mindfulness-based program completed by 30 first- and second-year medical students compared to wait-list controls. Participating students reported a decrease in stress level as well as enhanced mindfulness, self-compassion and sense of altruism. 88% of participants reported that the program had a positive impact on their academic performance and 69% reported a positive impact on clinical performance. [24] demonstrated that an 8-week mindfulness training program resulting in students developing a greater sense of control and resilience, which allowed them to better manage their workload and accept their limitations. Participants also communicated a sense of improved empathy and communication skills.

[25] was the only mandatory longitudinal mindfulness-based stress management intervention. The results did not show improvement in well-being among the 1st year medical students who participated. This study noted a decrease in quality of life and happiness, as well as an increase in stress for the participants, compared to previous cohorts. However, it was observed that one of the two cohorts demonstrated improved indicators of empathy.

Two studies evaluated shorter interventions. [26] assessed the effect of a 2-hour workshop intended to build mind-body and reflective writing skills. The Professional Quality of Life measure and a questionnaire evaluating the understanding of professional burnout and resilience was used to measure effectiveness of the intervention. Results demonstrated greater student awareness of burnout and resilience, as well as the development of meditation and reflective writing as coping strategies. [27] also assessed the use of a singular, mandatory session which taught 3rd and 4th year undergraduate medical students diaphragmatic breathing, self-control relaxation, and walking meditation. Participants reported a reduction of stress levels on the General Well-Being Scale, and a reduction in anxiety.

#### Non-Mindfulness-Focused Elective Interventions

[28] recruited 28 first- and second-year medical students to participate in seven equine-assisted therapy sessions over two months. Compared to baseline, there was a significant reduction in depression (p = <0.001), stress severity (p = 0.014), and stress frequency [p = 0.001). Students reported many benefits, including the opportunity to practice communication and teamwork skills which strengthened relationships.

[29] assessed the impact of an elective six-session theatre module for medical students. Students reported that the integration of a humanities elective was useful for enhancing overall well-being. Results demonstrated that the module was a source of fun, cultivated connections between peers, and enhanced personal growth and resilience.

The impact of physical activity and health promotion was assessed by [30] in a 6- week program using several rating scales. Mean scores on the WHO-5 Well-Being Index increased from 63.2 to 67.5 (p = < 0.01], sleep scale increased from 3.1 to 3.5 (p = < 0.001), and the loneliness scale decreased from 4.1 to 3.5 [p = < 0.005). Individuals who met standard guidelines for physical activity saw benefits in both physical and mental health, fostering greater resilience.

#### Reflection Groups

[31] assessed the effect of elective reflection groups, which were an opportunity for 1st and 2nd year medical students to share experiences, connect with peers, and learn emotional self-awareness skills. Participants reported improved well-being and self-awareness. [32] demonstrated similarly positive results from mandatory clinical debrief sessions in which 3rd year students shared cases and experiences during weekly small group discussions that took place over two consecutive 12-week blocks of the first clinical year. Students reported that the sessions provided a safe and structured environment for support and processing of emotions. Both studies suggested these interventions improved measures of empathy, and a greater sense of interpersonal connection.

#### Curricular Changes

[33] investigated stress levels directly related to the medical curriculum. The institution assessed perceived stress among students before and after a transition from a subject- to case-based curriculum. They noted an increased level of stress amongst students after the transition, with female students reporting higher levels of stress than males.

[34] also assessed the impact of curricular changes designed to improve mental health and academic success. This cohort-control study evaluated the effect of significant curricular changes including moving to a pass/fail system for preclinical courses, a 10% reduction of contact hours across the first 2 years of study, longitudinal electives, establishment of learning communities, as well as mandatory resilience and mindfulness programs. Students completed standardized assessments during their 1st year orientation and at the end of each year. The results showed an overall increase in satisfaction from 3.6 to 4.4 (on a 5- point rating scale] pre- to post curriculum changes, compared to the national mean of 3.9. They also noted a statistically significant increase (p = 0.002) in standardized medical test scores within the intervention group. Researchers found that curricular changes directly addressed common sources of stress including volume of material, level of detail of material, and competition for grades.

### Acceptability

Interventions were generally deemed acceptable to participants in the studies that assessed this domain. This was demonstrated through quantitative scales of satisfaction, and willingness to recommend to peers. The majority of participants in both the theatre and movement-based elective courses reported satisfaction and enjoyment [29,30]. The study of elective reflection groups by [31] illustrated satisfaction by participants who completed feedback surveys; however, attrition was high, cited by those who discontinued as being due to feeling overwhelmed by curricular activities.

## Discussion

This review demonstrates that a variety of types of interventions have been used to promote the well-being of undergraduate medical students, and that in general, these have had beneficial effects in domains of stress/burnout, resilience, mental health, academic performance, and interpersonal dynamics. Most interventions emphasized mindfulness skills, but other strategies also yielded beneficial outcomes.

All studies reviewed included an outcome measure related to perceived stress or burnout. Many also utilized assessments of resilience. Resilience may be thought of as the capacity of an individual to effectively cope with stressors, ultimately reducing the harmful impacts of persistent or intolerable stress [35]. Students who score high on perceived stress scales tend to score lower on scales of resilience [36], and students with lower levels of perceived stress tend to possess more resilience traits [37]. It follows then than in order to enhance a student’s ‘coping reservoir,’ an institution may offer a strategy to reduce sources of stress, and/or build skills of resilience. Given the demands of medical practice, building skills of resilience may be more important to prepare trainees for the reality of practice rather than reducing sources of stress. The balance is critical; too much stress with insufficient tools of resilience (a ‘toughen up’ model) is what can lead to burn-out through mechanisms of toxic stress [38]. Too little stress may prevent trainees from the incentive or opportunity to enhance their skills of resilience. [39] suggest that medical trainees are particularly vulnerable during early years of training as they adapt to their new environment, workload, and deadlines. Introducing interventions early in a student’s academic career seems to be important. Medical institutions may need to offer a progressive and flexible strategy to ensure all trainees have the opportunity to match growing sources of stress with growing skills of resilience. Many of the interventions reviewed also demonstrated a benefit to interpersonal dynamics and skills of empathy which is essential for healthcare providers. This would therefore not only add to student well-being, but potentially improve patient care as well in the future.

More specific programming may vary depending on available resources as well as the interests and needs of the particular student population. Involving the population for whom a program is being designed tends to optimize its relevance and effectiveness [40]. Only one of the studies reviewed [23] involved students in their program design and evaluation. Medical institutions would be wise to involve students to ensure strategies and intended outcome measures are appropriate to the population. For example, in 2016, the Doctor of Medicine programme at the University of Toronto initiated a creative resilience curriculum into their undergraduate medical program which aimed to support resilience throughout medical school, as well as decrease the stigma surrounding support [41]. Students were consulted on the creation and delivery of the curriculum and the results of both satisfaction surveys and focus groups showed value in the workshops. Ultimately, it was demonstrated that benefit was contingent on cooperation of faculty members, and demonstrated the need for collaboration to create a shift towards student wellness and resilience.

This review includes several mind-body or mindfulness interventions which were generally shown to reduce the impact of stress, improve psychological well-being, and are likely a feasible option in most institutions. However, while most participants in elective wellness-promoting programs report satisfaction, there is a strong risk of self-selection bias. Medical students experiencing stress, anxiety and depression tend to isolate themselves, or use other maladaptive coping strategies rather than seek out professional assistance [42]. Barriers to seeking help and participating in institutional programs include; stigma, cost, shortage of time, and fear of academic documentation [43]. Embedding well-being interventions within the curriculum has the potential to increase accessibility, reduce stigma, and engage students who might not otherwise choose to participate. [44] suggested mandated wellness programs may improve anxiety and perceived stress with no negative impact on academic performance, noting that students ‘reported enjoying sessions once trying them.’ Curriculum design changes may have positive effects on stress and burnout, while improving academic success [39]. However, it behooves medical institutions to consider unintended consequences of program-level, mandatory interventions [45]. Adding a mandatory course, or modifying the curriculum without providing adequate support for learners could have a detrimental effect, as seen in the studies by [25,33], or in the high attrition noted in the study by [31].

Although the results of this review were limited to undergraduate medical students, ensuring the well-being of all healthcare professionals and trainees is essential [46]. The COVID-19 pandemic in particular has shed light on the value and vulnerability of students [8,47,48], and the professionals they become [49]. As illustrated in Figure 1, promoting resilience, empathy, and constructive interpersonal skills among trainees can result in more resilient clinicians, cultivate positive working environments, and ultimately improve patient experience [50]. It would be in the best interests of society to embed curricular content and design which cultivates healthy coping mechanisms into the training of all health professionals.

### Strengths and Limitations

This review included a collection of studies using heterogeneous designs, interventions and outcome measures in an attempt to capture the scope of work done on this topic. However, doing so limited the ability to directly compare their effectiveness. Risk of bias was relatively high, as study quality was only fair to good for most. The lack of randomization, blinding or controls for many of the studies introduced systematic bias such as self-selection and contamination, therefore reducing internal validity. Most studies were single-centered and elective, reducing external validity. Most sample sizes were too small to determine statistical significance. Studies were not consistent with respect to the program length and year of study.

The search term of main interest was ‘well-being.’ This was deliberately selected to capture a wide range of interpretations. However, it is possible that it ultimately limited the results. Given the strong themes around resilience, it would be worthwhile for future reviews to specifically investigate this particular theme. Similarly, searching only PubMed likely limited the results and a broader environmental scan that included grey literature, conference proceedings, and other disciplinary databases may have revealed more content to analyze.

### Recommendations

Despite its limitations, this review suggests that when medical institutions offer strategies to reduce stress and promote well-being among undergraduate medical students, students tend to benefit. However, there are caveats. Given the prevalence of mental health struggles, as well as the stigma and other barriers reported by many medical students regarding seeking support, medical institutions would be wise to reduce barriers to participation. If curricular changes are being considered, they should be designed so as to minimize unnecessary strain while optimizing graduate competence. Given the emphasis in this review on the development of resilience skills and the value of reflection and peer connection, we are curious about the impact of the longitudinal ‘learning communities’ model which may provide benefits to student well-being [51].

Most importantly, we recommend that institutions and programs engage in a needs and resources assessment with their particular trainees to identify what is most needed and feasible in their setting. Strategies should be informed by evidence, and regularly reviewed and assessed. Quality improvement methodologies may be useful for institutions to assess their unique needs and design and evaluate potential strategies [52]. When evaluating strategies for knowledge dissemination, studies would be improved by including more rigorous controls, and directly comparing the difference in impact between variables such as the year of study, whether or not the intervention is voluntary, or directly comparing two different interventions within the same student population.

## Conclusion

Undergraduate medical students suffer from a higher prevalence of stress, anxiety, depression and substance abuse compared to other populations. When trainees participate in wellness-promoting interventions made available by their medical institution, they may benefit in the domains of mental health, resilience, academic performance, and interpersonal dynamics.

Strategies which aim to reduce unnecessary stress and cultivate resilience should be prioritized in all health professional education. This will strengthen the tenacity of healthcare professionals, which is reflected in improved quality of patient care. Further studies are required to identify which curriculum changes and programs are most effective and feasible for healthcare students in different settings, and to ultimately assess the impact on health system outcomes.

## References

[1] Yiu V. Supporting the well-being of medical students. Cmaj. 2005;172(7):889–13. 15795410 10.1503/cmaj.050126 PMC554874

[2] Nechita F, Nechita D, Pîrlog MC, et al. Stress in medical students. Rom J Morphol Embryol. 2014;55(3 Suppl):1263–1266. 25607418

[3] Zoccolillo M, Murphy GE, Wetzel RD. Depression among medical students. J Affect Disord. 1986;11(1):91–96. 2944933 10.1016/0165-0327(86)90065-0

[4] Dyrbye LN, Thomas MR, Shanafelt TD. Medical student distress: causes, consequences, and proposed solutions. Mayo Clin Proc. 2005;80(12):1613–1622. 16342655 10.4065/80.12.1613

[5] Dyrbye L, Shanafelt T. A narrative review on burnout experienced by medical students and residents. Med Educ. 2016;50(1):132–149. 26695473 10.1111/medu.12927

[6] Patel RS, Bachu R, Adikey A, et al. Factors related to physician burnout and its consequences: a review. Behav Sci. 2018;8(11):98. 10.3390/bs8110098 PMC6262585 30366419

[7] Hansell M, Ungerleider R, Brooks C, et al. Temporal trends in medical student burnout. Fam Med. 2019;51(5):399–404. 31081911 10.22454/FamMed.2019.270753

[8] Natalia D, Syakurah RA. Mental health state in medical students during COVID-19 pandemic. J Educ Health Promot. 2021;10. DOI:10.4103/jehp.jehp_1296_20 PMC8318147 34395645

[9] Hays LR, Cheever T, Patel P. Medical student suicide, 1989–1994. Am J Psychiatry. 1996;153(4):553–555. 8599405 10.1176/ajp.153.4.553

[10] Stern M, Norman S, Komm C. Medical students’ differential use of coping strategies as a function of stressor type, year of training, and gender. Behav Med. 1993;18(4):173–180. 8461489 10.1080/08964289.1993.9939112

[11] Supe AN. A study of stress in medical students at Seth GS Medical College. J Postgrad Med. 1998;44(1):1. 10703558

[12] Dunn LB, Iglewicz A, Moutier C. A conceptual model of medical student well-being: promoting resilience and preventing burnout. Acad Psychiatry. 2008;32(1):44–53. 18270280 10.1176/appi.ap.32.1.44

[13] Munn Z, Peters MDJ, Stern C, et al. Systematic review or scoping review? Guidance for authors when choosing between a systematic or scoping review approach. BMC Med Res Methodol. 2018;18(1):143. 30453902 10.1186/s12874-018-0611-x PMC6245623

[14] Arksey H, O’Malley L. Scoping studies: towards a methodological framework. Int J Soc Res Methodol. 2005;8(1):19–32.

[15] Bond AR, Mason HF, Lemaster CM, et al. Embodied health: the effects of a mind–body course for medical students. Med Educ Online. 2013;18(1):20699. 10.3402/meo.v18i0.20699 PMC3643075 23639275

[16] MacLaughlin BW, Wang D, Noone AM, et al. Stress biomarkers in medical students participating in a mind body medicine skills program. Evid Based Complement Alternat Med. 2011;2011:1–8. 10.1093/ecam/neq039 PMC3137844 21799696

[17] Kraemer KM, Luberto CM, O’Bryan EM, et al. Mind–body skills training to improve distress tolerance in medical students: a pilot study. Teach Learn Med. 2016;28(2):219–228. 27064724 10.1080/10401334.2016.1146605

[18] Williams MK, Estores IM, Merlo LJ. Promoting Resilience in Medicine: the Effects of a Mind–Body Medicine Elective to Improve Medical Student Well-being. Glob Adv Health Med. 2020;9:2164956120927367. 32499968 10.1177/2164956120927367 PMC7243374

[19] Xu YY, Wu T, Yu YJ, et al. A randomized controlled trial of well-being therapy to promote adaptation and alleviate emotional distress among medical freshmen. BMC Med Educ. 2019;19(1):1. 31159796 10.1186/s12909-019-1616-9 PMC6547604

[20] Yang E, Schamber E, Meyer RM, et al. Happier healers: randomized controlled trial of mobile mindfulness for stress management. J Altern Complementary Med. 2018;24(5):505–513. 10.1089/acm.2015.0301 29420050

[21] De Vibe M, Solhaug I, Tyssen R, et al. Mindfulness training for stress management: a randomised controlled study of medical and psychology students. BMC Med Educ. 2013;13(1):1. 23941053 10.1186/1472-6920-13-107 PMC3751423

[22] de Vibe M, Solhaug I, Rosenvinge JH, et al. Six-year positive effects of a mindfulness-based intervention on mindfulness, coping and well-being in medical and psychology students; Results from a randomized controlled trial. PloS one. 2018;13(4):e0196053. 29689081 10.1371/journal.pone.0196053 PMC5916495

[23] Danilewitz M, Bradwejn J, Koszycki D. A pilot feasibility study of a peer-led mindfulness program for medical students. Can Med Educ J. 2016;7(1):e31. PMC4830371 27103950

[24] Malpass A, Binnie K, Robson L. Medical students’ experience of mindfulness training in the UK: well-being, coping reserve, and professional development. 2019;Educ Res Int. 2019:1–10. 10.1155/2019/4021729 PMC6546595 31168420

[25] Dyrbye LN, Shanafelt TD, Werner L, et al. The impact of a required longitudinal stress management and resilience training course for first-year medical students. J Gen Intern Med. 2017;32(12):1309–1314. 28861707 10.1007/s11606-017-4171-2 PMC5698225

[26] Wald HS, Haramati A, Bachner YG, et al. Promoting resiliency for interprofessional faculty and senior medical students: outcomes of a workshop using mind-body medicine and interactive reflective writing. Med Teach. 2016;38(5):525–528. 27027210 10.3109/0142159X.2016.1150980

[27] Bughi SA, Sumcad J, Bughi S. Effect of brief behavioral intervention program in managing stress in medical students from two southern California universities. Med Educ Online. 2006;11(1):4593. 28253798 10.3402/meo.v11i.4593

[28] Chakales PA, Locklear J, Wharton T. Medicine and Horsemanship: the Effects of equine-assisted activities and therapies on stress and depression in medical students. Cureus. 2020;12(2). DOI:10.7759/cureus.6896 PMC7059872 32195063

[29] Nagji A, Brett-MacLean P, Breault L. Exploring the benefits of an optional theatre module on medical student well-being. Teach Learn Med. 2013;25(3):201–206. 23848325 10.1080/10401334.2013.801774

[30] Worobetz A, Retief PJ, Loughran S, et al. A feasibility study of an exercise intervention to educate and promote health and well-being among medical students: the ‘MED-WELL’programme. BMC Med Educ. 2020;20(1):1–2. 10.1186/s12909-020-02097-2 PMC7271428 32493427

[31] Gold JA, Bentzley JP, Franciscus AM, et al. An intervention in social connection: medical student reflection groups. Acad Psychiatry. 2019;43(4):375–380. 30963416 10.1007/s40596-019-01058-2

[32] Farrington R, Collins L, Fisher P, et al. Clinical Debrief: learning and well‐being together. Clin Teach. 2019;16(4):329–334. 31309726 10.1111/tct.13055 PMC6900240

[33] Ahmad FA, Karimi AA, Alboloushi NA, et al. Journal of dental education. Journal of Dental Education. 2017;81(5):534–544. 28461630 10.21815/JDE.016.026

[34] Slavin SJ, Schindler DL, Chibnall JT. Medical student mental health 3.0: improving student wellness through curricular changes. Acad Med. 2014;89(4):573. 24556765 10.1097/ACM.0000000000000166 PMC4885556

[35] Sisto A, Vicinanza F, Campanozzi LL, et al. Towards a Transversal Definition of Psychological Resilience: a Literature Review. Medicina (B Aires). 2019;55(11):745. 10.3390/medicina55110745 PMC6915594 31744109

[36] Thompson G, McBride RB, Hosford CC, et al. Resilience among medical students: the role of coping style and social support. Teach Learn Med. 2016;28(2):174–182. 27064719 10.1080/10401334.2016.1146611

[37] Shi M, Wang X, Bian Y, et al. The mediating role of resilience in the relationship between stress and life satisfaction among Chinese medical students: a cross-sectional study. BMC Med Educ. 2015;15(1):1–7. 25890167 10.1186/s12909-015-0297-2 PMC4332721

[38] McEwen BS. Neurobiological and Systemic Effects of Chronic Stress. Chronic Stress. 2017;1:2470547017692328. 10.1177/2470547017692328 PMC5573220 28856337

[39] Guthrie EA, Black D, Shaw CM, et al. Embarking upon a medical career: psychological morbidity in first year medical students. Med Educ. 1995;29(5):337–341. 8699970 10.1111/j.1365-2923.1995.tb00022.x

[40] Jull J, Giles A, Graham ID. Community-based participatory research and integrated knowledge translation: advancing the co-creation of knowledge. Implement Sci. 2017;12(1):1–9. 29258551 10.1186/s13012-017-0696-3 PMC5735911

[41] Kulman-Lipsey S, Yang S, Pedram Javidan A, et al. An integrative longitudinal resilience curriculum. Clin Teach. 2019;16(4):395–400. 31298474 10.1111/tct.13054 PMC6851571

[42] Chew‐Graham CA, Rogers A, Yassin N. ‘I wouldn’t want it on my CV or their records’: medical students’ experiences of help‐seeking for mental health problems. Med Educ. 2003;37(10):873–880. 12974841 10.1046/j.1365-2923.2003.01627.x

[43] Givens JL, Tjia J. Depressed medical students’ use of mental health services and barriers to use. Acad Med. 2002;77(9):918–921. 12228091 10.1097/00001888-200209000-00024

[44] Waechter R, Stahl G, Rabie S, et al. Mitigating medical student stress and anxiety: should schools mandate participation in wellness intervention programs? Med Teach. 2021;7:1. 10.1080/0142159X.2021.1902966 33832384

[45] Buja LM. Medical education today: all that glitters is not gold. BMC Med Educ. 2019;19(1):1. 30991988 10.1186/s12909-019-1535-9 PMC6469033

[46] Jennings BM. Work stress and burnout among nurses: role of the work environment and working conditions. Patient safety and quality: An evidence-based handbook for nurses; 2008. 21328768

[47] Chandratre S. Medical students and COVID-19: challenges and supportive strategies. J Med Educ Curric Dev. 2020;7:2382120520935059. 32637642 10.1177/2382120520935059 PMC7315659

[48] O’Byrne L, Gavin B, McNicholas F. Medical students and COVID-19: the need for pandemic preparedness. J Med Ethics. 2020;46(9):623–626. 32493713 10.1136/medethics-2020-106353 PMC7316103

[49] Chatzittofis A, Karanikola M, Michailidou K, et al. Impact of the COVID-19 pandemic on the mental health of healthcare workers. Int J Environ Res Public Health. 2021;18(4):1435. 33546513 10.3390/ijerph18041435 PMC7913751

[50] Oberg EB, Frank E. Physicians’ health practices strongly influence patient health practices. J R Coll Physicians Edinb. 2009;39(4):290. 21152462 10.4997/JRCPE.2009.422 PMC3058599

[51] Frosch E, Goldstein M. Relationship-Centered Advising in a Medical School Learning Community. J Med Educ Curric Dev. 2019;6:2382120519827895. 30937384 10.1177/2382120519827895 PMC6434435

[52] Wong BM, Headrick LA. Application of continuous quality improvement to medical education. Med Educ. 2021;55(1):72–81. 32790930 10.1111/medu.14351

